# Rosiglitazone-Mediated Effects on Skeletal Muscle Gene Expression Correlate with Improvements in Insulin Sensitivity in Individuals with HIV-Insulin Resistance

**DOI:** 10.4061/2011/736425

**Published:** 2011-04-12

**Authors:** Dennis C. Mynarcik, Margaret A. McNurlan, Mark M. Melendez, James A. Vosswinkel, Marie C. Gelato

**Affiliations:** ^1^Division of Endocrinology, Department of Medicine, Stony Brook University Medical Center, HSC T15-060, Stony Brook, NY 11794-8154, USA; ^2^Department of Surgery, Stony Brook University Medical Center, Stony Brook, NY 11794-8154, USA

## Abstract

Rosiglitazone, an agonist of peroxisome proliferator activated receptor (PPAR*γ*), improves insulin sensitivity by increasing insulin-stimulated glucose uptake into muscle tissue. This study was undertaken to assess changes in expression of PPAR-regulated genes in muscle tissue following treatment of HIV-associated insulin resistance with rosiglitazone. Muscle gene expression was assessed in twenty-two seronegative HIV subjects (control), 21 HIV-infected individuals with normal insulin sensitivity (HIV-IS) and 19 HIV-infected individuals with insulin resistance (HIV-IR). A subset of the HIV-IR group (*N* = 10) were re-evaluated 12 weeks after treatment with 8 mg/d of rosiglitazone. The HIV-IR group's rosiglitazone-mediated improvement in insulin sensitivity was highly correlated with increased expression of PPAR*γ* and carnitine palmitoyl transferase-1 (CPT-1), (*r* = 0.87, *P* < .001) and (*r* = 0.95, *P* < .001), respectively. The changes in PPAR*γ* expression were also correlated with the changes in CPT1 expression (*r* = 0.75, *P* = .009). The results suggest that rosiglitazone; may have a direct effect on muscle tissue to improve insulin sensitivity.

## 1. Introduction

With the increased use of multiple antiretroviral regimens including protease inhibitors, perturbations in glucose metabolism including insulin resistance and diabetes mellitus have been reported in patients infected with HIV (e.g., [1–5]). Insulin resistance predisposes individuals to diabetes, and insulin resistance and diabetes are also independent risk factors for cardiovascular disease [6–8]. Since HIV-infected individuals have additional risk factors for cardiovascular disease including hyperlipidemia (e.g., [9]) and increased visceral adiposity (e.g., [10]) with a demonstrably higher risk of myocardial infarction [11], several studies have investigated treatment of HIV-associated insulin resistance with antidiabetic drugs [12–17]. 

In treating HIV-associated insulin resistance, the thiazolidinedione, rosiglitazone has been used in several studies [12–16]. Rosiglitazone was selected not only because of its ability to improve insulin sensitivity, but also because of its potential to stimulate adipocyte differentiation [18] since the insulin resistance in HIV disease is associated with changes in body fat distribution including loss of adipose tissue from the peripheral subcutaneous regions [1, 19, 20]. In our small study [12] and those of others [13, 15, 16], improved insulin sensitivity with rosiglitazone treatment was associated with an improvement in body fat distribution, although altered body fat has not been reported in all studies (e.g., [21, 22]). 

 Rosiglitazone is a highly selective agonist for the gamma isoform of the peroxisome proliferator-activated receptor (PPAR) family of ligand-activated transcription factors [23, 24]. The expression of PPAR-*γ* is high in adipocytes and low in skeletal muscle. Because of the potential importance of PPAR*γ* in regulating adipogenesis and the higher expression of PPAR*γ* in adipocytes, Sutinen et al. examined adipose tissue gene expression in individuals with HIV-lipodystrophy treated for six months with PPAR*γ* agonist rosiglitazone. Only small changes in the expression of the PPAR*γ* and genes regulated by PPAR-*γ* such as PPAR-*γ* coactivator-1 (PGC-1), adiponectin, and IL-6 were observed, with no changes in other genes regulated by PPAR-*γ* including the genes for lipoprotein lipase, fatty acid binding protein or fatty acid translocases [25]. 

 Although PPAR-*γ* is only marginally expressed in skeletal muscle compared to adipose tissue, muscle is the primary tissue affecting peripheral glucose disposal [26]. Since rosiglitazone improved peripheral glucose disposal, as assessed by the hyperinsulinemic euglycemic clamp [12, 16], this study was undertaken to assess changes in gene expression in muscle tissue in individuals with HIV-associated insulin resistance treated with rosiglitazone. The expression of PPAR-responsive skeletal muscle genes was assessed from skeletal muscle biopsy specimens before and after 12 weeks of rosiglitazone administration. For comparison, the expression of PPAR-responsive skeletal muscle genes was also assessed in uninfected control subjects as well as HIV-infected subjects with and without impaired peripheral glucose uptake. 

## 2. Materials and Methods

### 2.1. Study Subjects

Gene expression was assessed using real-time PCR with an MJ research opticon instrument (Bio-Rad, Hercules, CA). RNA was extracted from muscle biopsies taken from 22 seronegative for HIV subjects (control), 21 HIV-infected individuals with normal insulin sensitivity (HIV-IS) and 19 HIV-infected individuals with insulin resistance (HIV-IR). Subjects were classified as insulin sensitive if peripheral glucose disposal from a hyperinsulinemic-euglycemic clamp [27] was greater than 1 SD below the mean for control subjects, that is, glucose disposal was less than 6 mg glucose per kg lean body mass per minute during an infusion of 1.2 mU insulin per kg body weight per minute. Muscle samples for gene expression analysis were available from only a subset of the HIV-IR group (*N* = 10) who took 8 mg/d rosiglitazone (4 mg of Avandia, Glaxo-SmithKline, Research Triangle Park, NC, USA. twice daily) for 12 weeks and studied again in the post-drug state. The exclusion criteria included an acute illness within 3 months preceding the study, a fasting glucose >126 mg/dl, a criteria eliminate individuals with hepatic insulin resistance or a random (nonfasting) blood glucose of >200 mg/dl, treatment for diabetes mellitus, or liver function tests in excess of three times normal values. 

 Subjects were monitored every 2 weeks for liver function tests, complete blood counts, CD4 counts, and viral load. In addition, blood pressure, weight, and physical appearance were also assessed at each visit. The study was approved by the Committee on Research involving Human Subjects at Stony Brook University and all subjects signed an informed consent form.

### 2.2. Insulin Sensitivity

Insulin sensitivity was determined as the rate of infused glucose necessary to maintain euglycemia during an infusion of insulin (hyperinsulinemic- euglycemic clamp) [27]. Subjects were admitted to the General Clinical Research Center the night before the study. The subjects were administered a defined snack (half a turkey sandwich) at 10 PM and were then fasted until completion of the procedure. At 7:00 a.m. the subjects were infused with 1.2 mU insulin (Humulin, Eli Lilly)/kg body weight per minute, sufficient to suppress hepatic glucose production in other insulin-resistant states [28]. Dextrose was administered intravenously at variable rates to maintain plasma glucose at 90 mg/dl. Plasma glucose was assessed from arterialized blood, obtained by the heated hand technique [29]. Insulin sensitivity was determined between the second and third hour of insulin infusion. To normalize for differences in body composition, insulin sensitivity is expressed as mg glucose/kg lean body mass (LBM) per minute. LBM was determined by dual energy X-ray absorptiometry (DEXA, model DPS, Lunar Radiation Co., Madison, WI) as previously described [1]. Insulin sensitivity data before and after rosiglitazone treatment was available for only 9 subjects and the improvement in insulin sensitivity with rosiglitazone has been reported previously [12]. 

### 2.3. Skeletal Muscle Biopsies

Skeletal muscle biopsies were obtained from the vastus lateralis, under sterile conditions using a 6 mm Bergstrom needle with vacuum assist. The vastus lateralis is a mixed fiber-type muscle composed both fast and slow twitch fibers, in relatively equal proportion, but the proportion is subject to perturbation as a function of the nature of exercise [30, 31]. The outer aspect of the thigh was shaved and cleansed using antiseptic soap. A small incision was used to expose the muscle surface and to allow the introduction of the needle. The vacuum was applied and the biopsy obtained. The biopsy specimen was quickly freed of excess blood, connective tissue and fat and frozen in liquid nitrogen. Pressure was applied until bleeding was stopped and the incision closed using sutures and steri strips. The frozen muscle tissue was subsequently ground to a powder under liquid nitrogen and stored in liquid nitrogen until processed for RNA extraction.

### 2.4. Gene Expression Analysis

Total RNA was extracted from frozen muscle powder using RNeasy lipid tissue minikit (Qiagen, Inc., Valencia, CA), according to the manufacturer's instructions. cDNA was prepared using SuperScript first-strand synthesis system with random hexamer priming (Invitrogen Corp., Carlsbad, CA). Gene expression was assessed using inventoried TaqMan gene expression assays, run with standard curves generated from plasmid DNA containing, either the I.M.A.G.E. clones, clones obtained through collaboration or a cloned PCR fragment spanning the amplicon used in TaqMan amplification. Inventoried kits for glyceraldehyde-3-phosphate dehydrogenase (GAPDH) Hs99999905_m1; lipoprotein lipase (LPL) Hs00173425_m1; muscle-type carnitinepalmitoyl transferase 1 (CPT-1) Hs00992651_g1; steroyl CoA-desaturase (SCD) Hs00748952_s1; adiponectin Hs00605917_m1 peroxisome proliferator-activated receptor-alpha (PPAR-*α*) Hs00231882_m1; PPAR-*γ* Hs00234592_m1; PPAR-*δ* Hs00602622_m1 and Δ9-cis retinoic acid receptor-alpha (RXR-*α*) Hs00172565_m1 and TaqMan master mix were obtained from Applied Biosystems (Foster City, CA). The sequences for the TaqMan assays are proprietary, and not available. I.M.A.G.E. clones for GAPDH (ID no. 3869809), LPL (ID no. 5768614), CPT-1 (ID no. 5245486), SCD (ID no. 3844850), PPAR-*α* (ID no. 6204635), PPAR-*γ* (ID no. 5198366), RXR-*α* (ID no. 3294824) were on obtained from OpenBiosystems (Huntsville, AL). The clone for human adiponectin was obtained from P. Scherer, Albert Einstein Medical College (Bronx, NY). The sequence for PPAR-*δ* was amplified from human adipose RNA using primers CCCCAAGCTTAGTACATGTCCTTGTAGATC and CCCGGAATCCATGGAGCA GCCACAGGAGG, digested with Bam H1 and Hind III and ligated into prepared pBluescript. All reactions were run in triplicate in 20 *μ*l volumes and the same samples were analyzed for the normalizing gene (GAPDH) on the same plate.

### 2.5. Statistical Analysis

The data are expressed as means ± SEM. Assessment of differences among groups was determined by ANOVA with Tukey adjustment for multiple testing. Assessment before and after treatment with rosiglitazone was made with paired *t*-test. Correlations were assessed by linear regression. Differences were considered significant if *P* < .05. All analyses were performed with SPSS (version 11.5 for Windows).

## 3. Results

The HIV-IR group had significantly lower skeletal muscle insulin sensitivity (4.4 ± 0.9 mg glucose per kg lean body mass per min, *P* < .001), assessed by the hyperinsulinemic- euglycemic clamp, than either the control group (10.6 mg ± 0.7) or the HIV-IS group (9.7 ± 0.6) (Table 1). 

**Figure 1:**
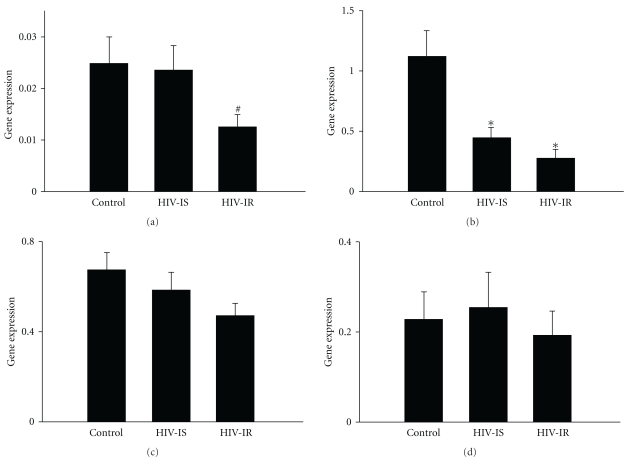
Gene expression for the gamma isoform of peroxisome proliferator-activated receptor (PPAR*γ*) (a), lipoprotein lipase (LPL) (b), carnitine palmitoyl transferase-1 (CPT-1) (c), and steroyl CoA-desaturase (SCD) (d) normalized to the expression of glyceraldehyde-3-phosphate dehydrogenase (GAPDH) in subjects without HIV disease (control) and subjects with HIV disease who were either sensitive to insulin (HIV-IS) or resistant to insulin (HIV-IR). Insulin sensitivity was determined with the hyperinsulinemic euglycemic clamp. Gene expression is expressed as mean with SEM and has been multiplied by 100. ^#^The expression of PPAR*γ* was significantly lower in the HIV-IP group than in the control group by simple *t*-test (*P* = .03), in (a). *The expression of LPL in both the HIV-IS and HIV-IR groups were significantly lower than controls (*P* < .01), in (b).

 Expression of the PPAR*γ* gene in muscle tissue was lower in HIV-infected subjects with insulin resistance (HIV-IR group) compared to uninfected subjects (control, Figure 1(a)) by simple *t*-test (*P* = .03). If the three groups, control, HIV-infected insulin sensitive (HIV-IS) and HIV-IR are compared with a correction for multiple testing, the differences among the groups are not significant. The expression of three PPAR-responsive genes, lipoprotein lipase (LPL), steroyl CoA-desaturase (SCD) and muscle-type carnitine palmitoyl transferase-1 (CPT-1) [32, 33], are also shown in Figure 1. The expression of lipoprotein lipase (LPL) was significantly reduced in both groups of HIV-infected subjects, relative to uninfected controls (*P* < .01, Figure 1(b)). The expression of muscle-type carnitine palmitoyltransferase 1 (CPT-1) was not significantly different among control, HIV-IS or HIV-IR groups (Figure 1(c)), and the expression of steroyl CoA-desaturase (SCD) was not different across groups (Figure 1(d)).

Rosiglitazone administration to HIV-infected subjects with insulin resistance significantly improved skeletal muscle insulin sensitivity, as previously reported for a subgroup of these subjects [12]. Treatment with rosiglitazone resulted in a small, but not significant (*P* = .08), increase in the expression of LPL (Table 2) and a significant increase (*P* = .03) in SCD expression (Table 2). There was a small (~20%) but significant (*P* = .018) decrease in CPT-1 expression (Table 2). Although the overall expression of PPAR*γ* was not significantly different before and after treatment with rosiglitazone (Table 2), this was mainly due to the variable response among individual subjects with a range of responses to rosiglitazone of 0.42–2.28 fold. Similarly, the average change in insulin sensitivity in response to rosiglitazone, 1.56 ± 1.05, included a range of response from 0.88–2.59 fold. 

**Figure 2:**
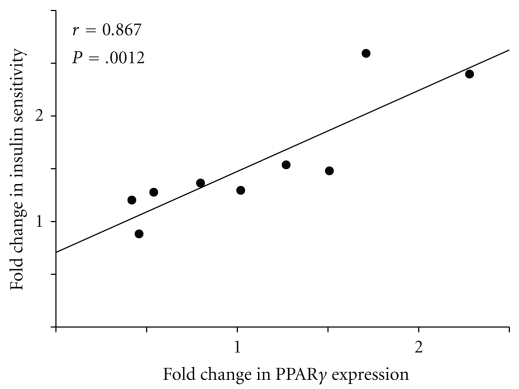
Relationship of changes in PPAR*γ* expression with changes in insulin sensitivity after rosiglitazone treatment. Correlation of the changes in the expression of the peroxisome proliferator-activated receptor-gamma (PPAR*γ*) gene normalized to the expression of glyceraldehyde-3-phosphate dehydrogenase (GAPDH) and the changes in insulin sensitivity in HIV infected subjects with insulin resistance before and after treatment with the PPAR*γ* agonist, rosiglitazone. The data are expressed as the fold changes and the correlation is highly significant (*r* = 0.87, *P* < .002).

The change in expression of the PPAR*γ* gene following rosiglitazone treatment was significantly correlated with the improvement in insulin sensitivity (Figure 2, *r* = 0.87, *P* < .002). The improvement in PPAR*γ* expression was also significantly associated with changes in expression of CPT-1 (*r* = 0.75, *P* < .009), but not LPL (*r* = 0.37, *P* = .30) or SCD (*r* = 0.11, *P* = .77). 

**Figure 3:**
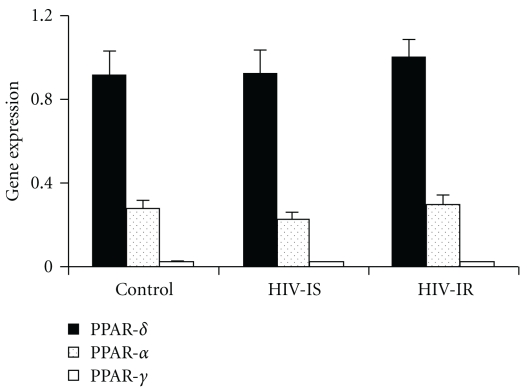
PPAR isoforms in skeletal muscle. Peroxisome proliferator-activated receptor PPAR isoforms (PPAR*δ*,  PPAR*α*,  PPAR*γ*) levels normalized to the expression of glyceraldehyde-3-phosphate dehydrogenase (GAPDH) in skeletal muscle in subjects without HIV infection (control) and subjects with HIV disease who were either sensitivity to insulin (HIV-IS) or resistant to insulin (HIV-IR). Insulin sensitivity was determined with the hyperinsulinemic euglycemic clamp. The data are expressed as mean and SEM multiplied by 100.

In addition, the low level of expression of PPAR*γ* in human vastus lateralis muscle compared to other isoforms of PPAR, that is, PPAR*α* and PPAR*δ*, was also confirmed. The level of the expression of PPAR*γ* was approximately 5% that of PPAR*δ* and 10% that of PPAR*α* (Figure 3).

To rule out the possibility of contamination of muscle biopsy specimens with adipose tissue, the expression of the adipose-specific gene, adiponectin, was assessed. Adiponectin expression in muscle tissue was 1000-fold lower than observed in adipose tissue in preliminary studies (data not shown). In addition, there was no change in adiponectin expression in the muscle specimens following treatment with rosiglitazone, as would be expected if there was significant contamination of the muscle specimens with adipose tissue. 

## 4. Discussion

Treatment of HIV-associated insulin resistance with a PPAR*γ* agonist (rosiglitazone) significantly improved insulin sensitivity assessed with the hyperinsulinemic euglycemic clamp. Hepatic gluconeogenesis is suppressed during the clamp by the infused insulin. The plasma glucose concentration is maintained at 90 mg/dl (5 mM) by infusion, functionally eliminating the hepatic GLUT2 transporters (Km 17 mM) from significantly contributing to glucose disposal. Thus, this procedure measures insulin sensitivity of peripheral tissue, primarily muscle [34]. The strong association of the improvement in insulin sensitivity with an increase in PPAR*γ* expression in muscle tissue following treatment with a PPAR*γ* agonist, rosiglitazone [23, 24], (Figure 2, *r* = 0.87, *P* = .001) suggests that a direct effect of rosiglitazone on muscle tissue may be important in mediating the improvement in insulin sensitivity. However, care must be taken to rule out an indirect effect on muscle biopsy material mediated by a direct effect on contaminating adipose tissue. 

Of the multiple isoforms of the PPARs, the expression of PPAR*γ* is much lower in muscle than either that of PPAR*α* or PPAR*δ* (Figure 3) and [35, 36]. In the present study, PPAR*γ* expression was approximately 10% that of PPAR*α* and only 5% of PPAR*δ* (Figure 3). The primary tissue expressing the PPAR*γ* isoform is adipose tissue with little expression in liver and muscle [37], suggesting that adipose tissue may be the primary site of action for the PPAR*γ* agonists like rosiglitazone. Of particular relevance to the present study is the ability of adipose tissue to secrete adiponectin in response to treatment with PPAR*γ* agonists such as rosiglitazone [38, 39] particularly a high-molecular-weight isoform [40, 41] associated with improved insulin sensitivity. 

However, the improvement in insulin sensitivity associated with increased adiponectin was associated with improvements in liver physiology, not muscle [40–43]. In the current study, insulin sensitivity was assessed with the clamp procedure, which reflects sensitivity in muscle tissue and not in the liver. 

The changes in the circulating concentrations of adiponectin including the concentration of the high-molecular weight isoform were not associated with the improvement in insulin sensitivity, as measured by the clamp. Therefore, it seems unlikely that the improvement in insulin sensitivity in the current study was mediated by an effect of rosiglitazone on adipose tissue resulting in the release of adiponectin with a subsequent effect on muscle tissue. 

An error in interpretation might also arise if there was significant contamination of the muscle biopsy with adipose tissue. To test for this possibility, the muscle samples were analyzed for the expression of the adiponectin gene, which is exclusively expressed in adipose tissue [44]. The level of expression of adiponectin in these muscle samples is approximately 1000-fold lower than in adipose tissue and completely unresponsive to the treatment with rosiglitazone (Table 2) making it most unlikely that the observed responses in PPAR*γ* gene expression were due to adipose tissue contamination of the muscle biopsies.

It seems likely, therefore, that the improvement in peripheral insulin sensitivity was related to a direct action of rosiglitazone on muscle tissue PPAR-regulated gene expression rather than an indirect effect mediated by adipose tissue. This conclusion is consistent with animal experiments indicating that treatment with PPAR*γ* agonists can act directly on muscle tissue [45]. The study of Kim et al. demonstrated that adipose tissue was not necessary for rosiglitazone-mediated improvement in insulin sensitivity. Glucose uptake into muscle was normalized following rosiglitazone treatment of the insulin-resistant A-ZIP/F-1 fatless mouse, although hepatic insulin resistance was not corrected, suggesting that a normalization of hepatic insulin resistance did require an adipose tissue-derived factor [45]. Development of a mouse model with a specific deletion for muscle PPAR*γ* also suggested that improvement in insulin sensitivity by PPAR*γ* agonists (both troglitazone and rosiglitazone) required the presence of muscle PPAR*γ* [46]. Other studies in humans have also confirmed the presence of PPAR*γ* in muscle tissue both with immumohistochemistry [47] and with RT-PCR [48, 49] adding additional support for the hypothesis of a direct effect on PPAR*γ* in muscle tissue.

The present study also assessed the expression of genes known to be regulated by PPAR*γ* in muscle tissue, including muscle-type carnitine palmitoyl transferase-1 (CPT-1), lipoprotein lipase (LPL), and steroyl CoA-desaturase (SCD) [18, 50] in response to treatment with a PPAR*γ* agonist, rosiglitazone. Treatment with rosiglitazone resulted in a significant stimulation of SCD expression (Table 2) which is consistent with an increase in SCD expression in muscle samples reported by Tonelli et al. for pioglitazone (a different PPAR*γ* agonist) treatment of subjects with type 2 diabetes [40]. In the present study there was also a small, but significant, decrease in CPT-1 and no significant change in the expression of LPL. The change in CPT-1 expression correlated with the change in PPAR*γ* expression (*r* = 0.95, *P* < .001), but the changes in expression of SCD (*r* = 0.11, *P* = .8) and LPL (*r* = 0.37, *P* = .3) were not significantly correlated with changes in PPAR*γ*. Thus, there is not an apparent generalized increased expression in PPAR*γ*-regulated genes in muscle tissue in response to treatment with a PPAR*γ* agonist, but rather selective regulation of PPAR*γ*-regulated genes. 

Although the relationship of changes in PPAR*γ* expression with changes in expression of CPT-1 following treatment were highly significant (*r* = 0.95, *P* < .001), overall the expression of CPT-1 was lower after treatment than before. This somewhat paradoxical result has also been reported in a study in diabetic Zucker rats treated with a PPAR*γ* agonist (GW1929). In that study a number of differences in response between adipose tissue and muscle tissue in response to treatment with a PPAR*γ* agonist were noted with the expression of multiple genes involved in fatty acid transport and oxidation increased in adipose tissue but decreased in muscle tissue [33]. A somewhat different conclusion was reported by Wilmsen et al. in human muscle cells from diabetic subjects in culture exposed to rosiglitazone [51] where treatment of the cultures with a series of PPAR*γ* agonists resulted in increased uptake and oxidation of fatty acids. This in vitro result was not seen in an in vivo study of rats treated with a PPAR*γ* agonist [52]. 

The present study also suggests that there is defective gene expression associated with HIV-infection whether or not insulin resistance is present. The expression of lipoprotein lipase (LPL, Table 2) was significantly reduced in those individuals infected with HIV regardless of their insulin sensitivity. Although a correlation of human skeletal muscle LPL expression with PPAR*α* expression has been reported [53], it is unlikely that the reduced expression of LPL in subjects infected with HIV was due to lower levels of PPAR*α* levels. PPAR*α* expression was not reduced in HIV-insulin sensitive individuals compared to control subjects (Figure 3). It seems more likely that the reduction in LPL expression with HIV disease was due to factors other than PPAR-regulation of the LPL promoter region. 

 In conclusion, this study suggests that human muscle tissue may be responsive to the effects of the PPAR*γ* agonist, rosiglitazone, and that the changes in expression of the PPAR*γ* gene in muscle tissue may be a contributing factor to the improvement in insulin sensitivity observed following rosiglitazone administration (Figure 2). 

##  Conflict of Interests 

The authors have no conflict of interests.

## Figures and Tables

**Table 1:** Subject characteristics.

	Control	HIV-IS	HIV-IR	HIV-IR (treated)
	(*N* = 22)	(*N* = 21)	(*N* = 19)	(*N* = 10)
Gender (M/F)	10/12	13/8	8/11	4/6
Race/ethnicity (AA/H/C)	2/2/18	15/2/4	10/3/6	7/0/3
Age (y)	38 ± 1.5	41 ± 1.4	41 ± 1.5	41 ± 2.3
BMI (kg/m^2^)	25.5 ± .9	28 ± 1.4	27 ± .8	26 ± 1.0
Viral load (copies/mL)	N/A	400 (40; 34,320)	50 (40; 16,291)	900 (40; 16,291)
CD4+ cells (cells/mm^3^)		500 ± 65.3	530 ± 58.8	668 ± 73.8
PI, NRTI, NNRTI		2	2	1
PI, NRTI		12	7	4
NRTI, NNRTI		4	5	2
NRTI		0	1	0
No HIV meds		3	3	2
Triglycerides (mg/dL)	104 ± 10.5	132 ± 14	324 ± 79	259 ± 57
Insulin sensitivity (mg glucose/kg LBM/min)	10.6 ± 0.74	9.7 ± 0.43	4.4 ± 0.90*	4.3 ± 0.3

Values are means ± SEM except viral load which is expressed as median and range. AA: African American; C: Caucasian; H: Hispanic; PI: protease inhibitor; NRTI: nucleoside reverse transcriptase inhibitor; and NNRTI: nonnucleoside reverse transcriptase inhibitor. HIV-IS: insulin sensitivity HIV subjects. HIV-IR: insulin-resistant HIV subjects. HIV- IR (treated) denotes subjects with insulin resistance who were treated with rosiglitazone (8 mg/d for 12 weeks). Insulin sensitivity was determined with the hyperinuslinemic euglycemic clamp and is expressed as mg glucose per kg per lean body mass per minute. *Insulin sensitivity in the HIV-IR group was lower than either the control or the HIV-IS group (*P* < .001). The data on improvement in HIV-associated insulin resistance with rosiglitazone treatment has been previously reported [12].

**Table 2:** Gene expression in muscle tissue of insulin-resistant HIV infected subjects before and after treatment with rosiglitazone.

	Before rosi	After rosi	Fold change	*P* value
	(×10^−4^)	(×10^−4^)		
LPL	24.7 ± 2.8	30.2 ± 4.8	1.27 ± 0.16	NS
CPT-1	57.6 ± 5.3	44.7 ± 4.3	0.81 ± 0.08	.018
PPAR*γ*	0.954 ± 0.177	0.942 ± 0.185	1.12 ± 0.21	NS
ACPR30	4.14 ± 2.1	5.13 ± 2.6	1.76 ± 0.58	NS
SCD	16.3 ± 7.2	25.3 ± 9.6	1.67 ± 0.27	.03
PPAR*α*	34.5 ± 6.2	35.2 ± 5.0	1.17 ± 0.14	NS

All gene expression data before and after treatment with rosiglitazone (8 mg/d for 12 weeks) are expressed as means ± SEM and normalized to the expression of glyceraldehyde-3-phosphate dehydrogenase (GAPDH). LPL: lipoprotein lipase; CPT-1: carnitine palmitoyl transferase-1; PPAR*γ:* peroxisome proliferator-activated receptor *γ*; ACPR30: Adiponectin; SCD: steroyl CoA-desaturase; and PPAR*α:* peroxisome proliferator-activated receptor *α*. Statistical significance was determined with a paired *t*-test (SPSS).
